# Trehalose Outperforms Chitosan, Humic Acid and Gamma-Aminobutyric Acid in Promoting the Growth of Field Maize

**DOI:** 10.3389/fpls.2022.889615

**Published:** 2022-06-14

**Authors:** Bingyan Li, Tengfei Guo, Wei Zhou

**Affiliations:** ^1^Institute of Agricultural Resources and Regional Planning, Chinese Academy of Agricultural Sciences, Beijing, China; ^2^Institution of Plant Nutrition and Environmental Resources, Henan Academy of Agricultural Sciences, Zhengzhou, China

**Keywords:** plant growth regulator, endogenous hormones, carbon assimilation, nitrogen metabolism, maize

## Abstract

Despite the fact that there are many distinct types of plant growth regulators (PGRs), the diverse ways in which they regulate plant development are rarely compared. In this study, four PGRs (trehalose, chitosan, humic acid and gamma-aminobutyric acid) were selected and sprayed folially, and plant samples were collected while maize was at vegetative leaf stages 6, 10, and 14 (V6, V10, and V14, respectively) to reveal the effects of different PGRs on photosynthesis, dry matter accumulation, oxidative stress, carbon and nitrogen metabolism, hormone levels, and gene expression of maize. Results showed that 100 mg/L PGRs did not induce oxidative damage or repair activities in maize. Trehalose significantly increased chlorophyll content at V6 and promoted dry matter (roots and shoots) accumulation at V6 and V10. The activities of carbon and nitrogen metabolizing enzymes were significantly enhanced by trehalose treatment, which promoted the accumulation of sucrose and soluble sugar, but did not affect the biosynthesis of auxin and gibberellin at V6. Changes in carbon and nitrogen metabolism enzymes are regulated by transcription of related synthetic genes. Lower starch content and higher sucrose content in trehalose-treated maize leaves are important biological characteristics. Further analysis revealed that the effect of trehalose on the metabolic activity of maize was a short-term promoting effect (0–12 days), while the effect on maize growth was a long-term cumulative effect (13–28 days). Overall, exogenous trehalose induced stronger carbon and nitrogen metabolic activity, higher photosynthetic capacity and more dry matter accumulation than chitosan, humic acid and gamma-aminobutyric acid.

## Introduction

Global climate change is gradually transforming agricultural production and development patterns. Maize is an important source of food, feed and biofuel and the most widely grown cereal crop in global agricultural production (Zhang et al., 2017). The Food and Agriculture Organization (FAO) has projected that the global population will grow from 7.6 billion at present to 9 billion by 2050 (Bahar et al., 2020). Rapid population growth has led to increased demand for food, and the imbalance between supply and demand has gradually become an acute problem. How to improve plant growth, promote crop yield and reduce pollution has become an important research focus of scientists. Sustainable field management practices, targeted gene editing techniques and application of nitrogen-fixing microorganisms and plant growth regulators (PGRs) are particularly effective strategies (Siddiqui et al., 2022). PGRs are natural products of small molecules that improve plant growth at very low concentrations, including trehalose, chitosan, humic acid, and gamma-aminobutyric acid, among others (Zhang et al., 2022).

Trehalose is a non-reducing disaccharide that can be synthesized by all organisms except vertebrates (Paul, 2007). It acts as a molecular stabilizer in higher plants and as a carbon source in lower organisms (Elbein et al., 2003). Application of exogenous trehalose and endogenous overexpression of trehalose can enhance the resistance of plants to drought, salt damage, low temperature and other stresses by promoting carbohydrate synthesis, increasing the activity of antioxidant enzymes, regulating changes in hormone levels and reducing ROS levels (Ali and Ashraf, 2011; Abdallah et al., 2016; Zulfiqar and Ashraf, 2021). Accordingly, the combined application of trehalose and salicylic acid to sweet basil (*Ocimum basilicum* L.) was more effect than application of trehalose alone in improving drought resistance, promoting plant growth and increasing dry matter accumulation (Zulfiqar et al., 2021). Chitosan is an abundant natural biopolymer and a product of chitin deacetylation, and the complex diversity of structure and function of chitosan expands its application in agriculture. Chitosan nanomaterials can inhibit the incidence of *Fusarium oxysporum* (Elsherbiny et al., 2022) or increase maize yield by increasing chlorophyll content and leaf area index (Kumaraswamy et al., 2021). Chitosan oligomers have also been shown to improve the resistance of maize to cadmium stress (Qu et al., 2019). Chitosan and calcium carbonate can mitigate the negative effects of salt stress on wheat (Sadak and Talaat, 2021). Silicon and chitosan have been shown to improve maize resistance to salt stress (Younas et al., 2021). Humic acid is the main extractable part of humic compounds with hormone-like activity [e.g., auxin (IAA) and gibberellin (GA)] and is a natural chelating agent (Nikbakht et al., 2008). Foliar spraying of humic acid improved rapeseed oil yield and quality by promoting nutrient uptake and transport (Nasiri et al., 2021). Humic acid application improved soybean seedling tolerance (Matuszak-Slamani et al., 2017). The four-carbon non-protein amino acid gamma-aminobutyric acid is distributed among animals, plants and microorganisms (Kinnersley and Lin, 2000). Because gamma-aminobutyric acid production can be rapidly induced under biotic and abiotic stresses in response to various environmental signals. Recent studies have shown that gamma-aminobutyric acid is involved in signal transduction and regulation of carbon and nitrogen metabolism balance in higher plants, reduces oxidative damage in plants, improves plant stress resistance and mediates the interaction between plants and microorganisms (Ramesh et al., 2017).

However, the majority of their conclusions are based on laboratory experiments, with only a small amount of research on the effects of field circumstances on maize growth. The vegetative growth period is a time during which plants are particularly sensitive to environmental changes. Accordingly, we investigated photosynthesis, carbon and nitrogen metabolism, endogenous hormones, oxidative stress and key metabolic genes of maize at three growth stages [vegetative leaf stages 6, 10, and 14 (V6, V10, and V14, respectively)] under foliar-spraying of PGRs. Pearson correlation analysis is effective in revealing complex metabolic regulatory activities. In addition, the application of principal component analysis and partial least squares discriminant analysis (PCA and PLS-DA) were utilized to better distinguish the significant differences between different treatments. Thus, this study was undertaken to evaluate the effects of different PGRs on maize growth and reveal their differential regulatory mechanisms, and the findings have significant implications for the use of PGRs in agricultural production.

## Materials and Methods

### Experimental Design

The experiment site was located in Ping yuan New District, Xinxiang City, Henan Province, China (113°40′42″E, 34°47′55″N). The soil type in this area is fluvo-aquic soil, and the average annual rainfall is 645 mm, mainly concentrated in the 3 months of July, August and September. The annual evaporation is 1,450 mm, and the annual average temperature is 14.4°C. The maize variety selected for the present study was “Jun dan 20” (cultivated by the Institute of Agricultural Sciences of Jun xian County, Henan Province, China). The amount of fertilizer applied during the season was based on the recommended amount of fertilizer for summer maize, consisting of N (210 kg/hm^2^), P_2_O_5_ (75 kg/hm^2^) and K_2_O (90 kg/hm^2^). The experimental plot was divided into subplots, each 6 m × 8 m, with a total of 15 subplots. The experimental treatment was based on a random block design.

Untreated water was the control (because water was not expected to negatively affect plants) treatment. The other treatments included addition of the PGRs trehalose (Sigma Aldrich Shanghai Trading Co., Ltd., Shanghai, China), chitosan (Shanghai Aladdin Biochemical Technology Co., Ltd., Shanghai, China), humic acid (Sigma Aldrich Shanghai Trading Co., Ltd.) and gamma-aminobutyric acid (Beijing Solai Bao Technology Co., Ltd., Beijing, China), respectively. Each treatment included three replicates. The maize sowing date was June 8, 2021, the sowing depth was 7 cm, and each plot was planted in 10 rows. The field management measures after sowing were conducted in accordance with the local farmland production practices. The 100 mg/L PGR treatments were applied by foliar spraying. The spraying was conducted before sunset in the evening, and 0.1% Tween 20 was added to the solvent. Plots were sprayed on June 18, June 23 and June 28, three times in total. Each plant was sprayed with 10 mL (in order to ensure the consistency and effectiveness of the treatment).

The sampling points of plant samples were June 30 (V6), July 16 (V10) and August 3 (V14), and these three growth points were selected because they are the key nodes in the vegetative growth of maize (Bender et al., 2013). A portion of the collected maize leaves (the middle of the leaf, with the main veins removed) were stored at –20°C for the subsequent detection of physiological and biochemical indexes; the other portion was stored at –80°C for subsequent assays of the composition of samples. Additionally, five whole plants were collected for the calculation of the accumulation of maize biomass. Roots were collected from a soil volume of 30 × 30 × 35 cm (length × width × height).

### Detection Methods

#### Photosynthetic Index

Complete functional leaves of similar shape and size were selected, and a SPAD 502 DL Plus instrument (Konica Minolta, Tokyo, Japan) was used to measure the leaves at the center of maize plants under different PGR treatments at V6, V10 and V14.

#### Oxidative Stress Indicators

For all assays of oxidative stress indicators, 0.1-g samples of maize leaves were added to 1 mL of PBS buffer (Phosphate buffered saline, 0.01 M, PH 7.2–7.4), homogenized with a pre-cooled mortar on ice and centrifuged at 10,000 × *g* for 10 min at 4°C, and the resulting supernatant was collected for later use.

##### Determination of Malondialdehyde Content

First, 1 mL of 0.5% thiobarbituric acid was added to the supernatant, which was mixed well and placed in boiling water for 10 min. The content of lipid peroxide was estimated by measuring the absorbance at 532 nm and the absorbance value at 600 nm; the difference between the two values was used for the calculation of malondialdehyde (MDA) content.

##### Determination of the Total Antioxidant Capacity

First, 0.18 mL of FRAP working solution (0.3 M acetate buffer:10 mmol/L TPTZ:20 mmol/L FeCl_3_, 10:1:1, *V*:*V*:*V*) was added to the supernatant, which was then incubated in a water bath at 37° for 10 min. Then, the absorbance at 593 nm was measured, and the depth of the color reflected the total antioxidant capacity (T-AOC) of the plant. Distilled water was used to determine the zero value, and the result was recorded as units per gram (U/g).

#### Carbon and Nitrogen Metabolites

The soluble sugar, sucrose, NO_3_^–^ and starch contents were measured using the methods of Hu et al. (2021). Free amino acid (FAA) content was determined by utilizing the principle that amino acids and ninhydrin can be quantified when they are co-heated. In short, fresh plant leaves were washed, cut into pieces and mixed evenly. Then, 0.1-g samples were added to 10 mL of distilled water and placed in a boiling water bath for 20 min. Supernatants were transferred to 20-mL scale test tubes, and a small amount of distilled water was continually added after repeated extraction of a constant volume. Then, 0.5 mL of NaCN buffer and ninhydrin hydrate was successively added, and after 12 min in a boiling water bath, 5 mL of 95% ethanol was added. The absorbance value was measured at a 570 nm wavelength for the calculation of the amino acid concentration.

#### Carbon and Nitrogen Metabolism Enzymes

The enzymes involved in carbon metabolism, including sucrose synthase (SS), ADPG pyrophosphorylases (AGPases), sucrose phosphate synthase (SPS), isocitrate dehydrogenase (IDH), phosphoenolpyruvate carboxylase (PEPC) and 1,5-ribulose diphosphate carboxylase (Rubisco) were measured using the corresponding assay kits following the manufacturer’s instructions (Beijing Chejeter Technology Co., Ltd., Beijing, China). The activities of enzymes related to nitrogen assimilation, including glutamate dehydrogenase (GDH), nitrate reductase (NR), glutamate synthase (GS), and glutamine synthase (GOGAT), were determined according to the assay kit instructions (Qi Yi Biological Technology Co., Ltd., Shanghai, China).

#### Endogenous Plant Hormones

The contents of IAA and GA were determined using an ELISA kit (Qi Yi Biological Technology Co., Ltd.). First, 0.1-g samples of maize leaves were added to 1 mL of PBS buffer to prepare the homogenate. Samples were centrifuged at 8,000 × *g* for 10 min, and 10 μL of supernatant was collected and added to 40 μL of sample buffer. Then, 100 μL of horseradish peroxidase (HPR)-labeled detection antibody was added, and samples were incubated in a water bath at 37° for 60 min. Substrate for the chromogenic reaction assay was added after the samples were washed, and after incubation for 15 min away from light, stop solution was added. The hormone content was calculated by measuring the absorbance at 450 nm.

#### Quantitative Reverse Transcription PCR

The extraction of RNA was accomplished with a plant total RNA extraction kit. RNA extraction quality and concentration were assessed by spectrophotometry and agarose gel electrophoresis. The reverse transcription program was as follows: 42°C for 15 min and 95°C for 3 min. RNA was reverse transcribed, and gene expression was detected using the SYBR Green kit (Tian gen Biotech Co., Ltd., Beijing, China). Quantitative reverse transcription PCR (RT-qPCR) assays were performed on Applied Biosystems ABI ViiATM7 platform (Applied Biosystems, Waltham, MA, United States). The upstream and downstream primers were designed using Primer Premier 5.0 software (Premier Biosoft Int., Palo Alto, CA, United States), and the sequence information of the target gene was obtained from NCBI^1^ (Supplementary Table 1). The following PCR amplification program was used: Pre-denaturation at 95°C for 15 min, followed by 40 cycles of denaturation at 95°C for 10 s, annealing at 58°C for 20 s and extension at 72°C for 32 s. β*-actin* was used as an internal reference gene, and the relative expression of the target gene was calculated using the 2^–ΔΔCT^ method.

### Data Analysis

The data presented in all graphs are mean ± standard error (SE) values, and statistical analysis was conducted with SPSS Statistics 23 (IBM Corp., Armonk, NY, United States). One-way ANOVA was used to determine significant differences between treatments, and the significance level applied was *P* < 0.05.

## Results

### Oxidative Stress State

First, to assess whether foliar spraying of PGRs can alter the growth status of planted maize, levels of oxidative damage and T-AOC were analyzed.

Trehalose, chitosan, humic acid, and gamma-aminobutyric acid did not significantly change the content of MDA, and T-AOC of maize compared with the control conditions at V6, V10 and V14 (Table 1).

**TABLE 1:** Effects of PGR treatments on oxidative stress in maize.

Indexes	Treatment	Growth period		
		V6	V10	V14
MDA (nmol/g FW)	Control	28.64 ± 2.73	23.52 ± 1.54	25.89 ± 2.10
	Trehalose	30.37 ± 1.65	24.79 ± 3.48	29.41 ± 0.50
	Chitosan	26.64 ± 0.66	22.88 ± 3.27	27.98 ± 3.30
	Humic acid	26.24 ± 2.55	27.61 ± 2.19	30.07 ± 4.79
	Gamma-aminobutyric acid	25.42 ± 1.60	23.77 ± 2.94	23.21 ± 4.75
T-AOC (U/g FW)	Control	16.57 ± 1.37	11.50 ± 1.00	11.13 ± 0.04
	Trehalose	14.73 ± 2.00	11.18 ± 1.43	11.47 ± 2.35
	Chitosan	16.50 ± 1.72	10.74 ± 0.24	12.38 ± 1.42
	Humic acid	15.88 ± 2.27	9.83 ± 0.61	10.36 ± 0.68
	Gamma-aminobutyric acid	17.03 ± 2.14	11.74 ± 1.35	9.03 ± 0.58

*Statistical analysis was used to compare differences in means between PGR treatments shown. Within each stage, treatments labeled with different lowercase letters were significantly different (P < 0.05; n = 3), and the lack of such letters indicates the lack of a significant difference. V6, V10, and V14 represent the 6-, 10-, and 14-leaf stages of vegetative growth, respectively. MDA, malondialdehyde; T-AOC, total antioxidant capacity; FW, fresh weight.*

### Photosynthetic Capacity and Plant Traits

We detected changes induced by PGRs in the chlorophyll content of maize leaves at three growth stages (V6, V10, and V14). However, we found that compared with the control conditions at V6, only the chlorophyll content after trehalose treatment was significantly increased by 6.85%, while chitosan, humic acid, and gamma-aminobutyric acid treatments induced no significant differences. There was no significant difference between the groups treated with PGRs at V10 and V14 (Supplementary Figure 1).

**FIGURE 1:**
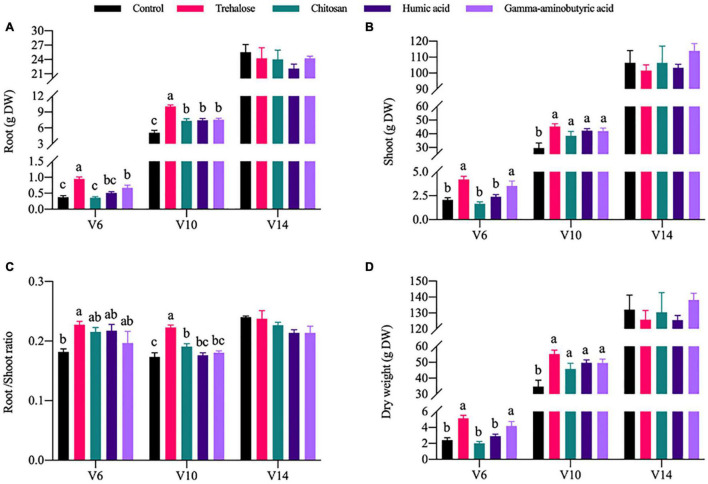
Effects of PGRs on maize growth at different growth stages. Comparisons were made between PGRs treatments with different colors in the same growth period; significant differences are indicated by different lowercase letters in the same figure panel, and no letter indicates no significant difference (*P* < 0.05, *n* = 5). V6, V10, and V14 represent the 6-, 10-, and 14-leaf stages of vegetative growth, respectively. DW, dry weight of the plant. **(A)** Root. **(B)** Shoot. **(C)** Root/shoot ratio. **(D)** Dry weight.

In contrast with the chlorophyl content results, growth of the maize plants was significantly promoted by PGR treatments. The trehalose and gamma-aminobutyric acid treatments were most significant at V6 and V10. Compared with the control conditions at V6, the dry weight of root tissue (Figure 1A), shoot tissue (Figure 1B) and the whole plant (Figure 1D), were significantly increased under trehalose treatment by 1.52, 1.04, and 1.12 times, respectively, and the dry weight of root tissue, shoot tissue and the whole plant was significantly increased under the gamma-aminobutyric acid treatment by 0.78, 0.71, and 0.72 times, respectively. All PGR treatments improved the growth of maize compared to the control conditions at V10. In addition, we analyzed the change in the maize root-to-shoot ratio (Figure 1C) under various PGR treatments. In short, our results showed that trehalose is the PGR that leads to the most significant accumulation of maize dry matter.

### Carbon Metabolism Enzymes and Carbon Metabolites

**FIGURE 2:**
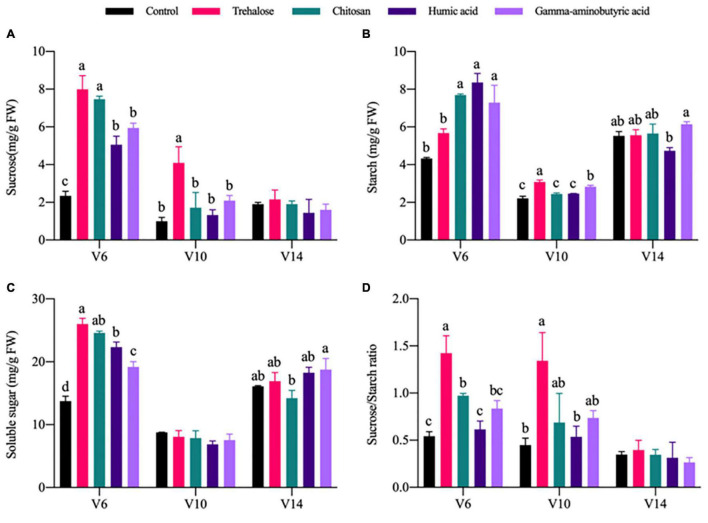
Effects of PGRs on carbon metabolite accumulation in maize at different growth stages. Statistical analysis was used to compare differences in the mean among the PGR treatments shown in different colors. Treatments labeled with different lowercase letters were significantly different (*P* < 0.05; *n* = 3), and the lack of such letters indicate no significant difference among treatments at each stage. V6, V10, and V14 represent the 6-, 10-, and 14-leaf stages of vegetative growth, respectively. FW, fresh weight. **(A)** Sucrose. **(B)** Starch. **(C)** Soluble sugar. **(D)** Sucrose/starch ratio.

Carbon metabolic activity is related to changes in plant photosynthetic capabilities. Compared with control conditions at V6, all PGRs promoted varying degrees of carbon metabolite accumulation. Among metabolite response variables, the sucrose content (Figure 2A), soluble sugar content (Figure 2C) and sucrose-to-starch ratios (Figure 2D) of trehalose treatment were significantly increased by 2.4, 0.89, and 1.63 times, respectively, but the change in starch content (Figure 2B) was not significant. Sucrose, starch and soluble sugar contents and the sucrose-to-starch ratios under chitosan treatment were significantly improved by 2.18, 0.78, 0.79, and 0.8 times, respectively. The sucrose, starch and soluble sugar contents under treatment with humic acid were significantly improved by 1.15, 0.93, and 0.63 times, respectively. Changes under gamma-aminobutyric acid treatment were basically consistent with those under humic acid treatment.

The synthesis of carbon metabolites is rigorously regulated by enzyme activity or content (Supplementary Figure 2). Rubisco, SPS, SS and PEPC activity levels were significantly improved by trehalose, chitosan and gamma-aminobutyric acid treatments. Humic acid treatment significantly improved AGPase and PEPC activity. The activity of IDH was improved in all PGRs, but only trehalose treatment induced significant differences at V6, V10 and V14.

### Nitrogen Metabolism Enzymes and Nitrogen Metabolites

**FIGURE 3:**
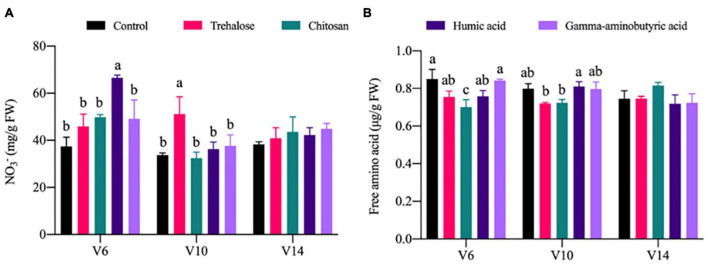
Effects of plant growth regulators (PGRs) on nitrogen metabolite accumulation in maize at different growth stages. Statistical analysis was used to compare means of PGR treatments shown in different colors; significantly different treatments within each stage are labeled with different lowercase letters (*P* < 0.05; *n* = 3), and the lack of such letters indicates there was no significant difference. V6, V10, and V14 represent the 6-, 10-, and 14-leaf stages of vegetative growth, respectively. FW, fresh weight.

The effects of PGR treatments on the accumulation of nitrogen metabolites were weak overall, but differed individually. Compared with the control conditions at V6, the NO_3_^–^ content under humic acid treatment was significantly increased by 0.78 times. Compared with the control conditions at V10, NO_3_^–^ content under trehalose treatment was significantly increased by 0.52 times. Other PGRs induced no significant difference under V6 and V10 (Figure 3A). Compared with the control conditions at V6, the content of FAA under chitosan treatment was significantly decreased by 0.18 times. There were no significant differences at V6 and V10 in the other PGR treatments (Figure 3B).

In contrast with the accumulation of nitrogen metabolites, the activity of nitrogen metabolic enzymes was actively regulated by PGRs. Compared with the control conditions at V6, the activity levels of NR, GOGAT and GS under trehalose treatment were significantly increased by 0.54, 0.57, and 0.28 times, respectively. The NR, GOGAT and GDH activity levels under chitosan treatment were increased by 0.44, 0.68, and 0.17 times, respectively. Other PGR treatments induced no significant difference compared with the control conditions (Supplementary Figure 3).

### Plant Hormone

Plant hormones are another important regulator of plant growth. GA and IAA differed in their response to treatments. The changes in GA and IAA induced by the application of PGRs were concentrated earlier (at V6 and V10) and later (at V10 and V14), respectively (Supplementary Figure 4).

Compared with the control conditions at V6, the content of GA was significantly increased by 0.1 times under treatment with chitosan, and there was no significant difference for other PGR treatments. In addition, all PGR treatments did not alter the level of IAA at V6. Compared with the control conditions at V10, humic acid resulted in significant increases of 0.16 and 0.1 times in GA and IAA, respectively, but gamma-aminobutyric acid only significantly increased GA content. Compared with the control conditions at V14, the effects of PGRs on the synthesis of GA and IAA were not significantly different, but the accumulation of IAA differed between treatments with different PGRs.

### Pearson Correlation Analysis

**FIGURE 4:**
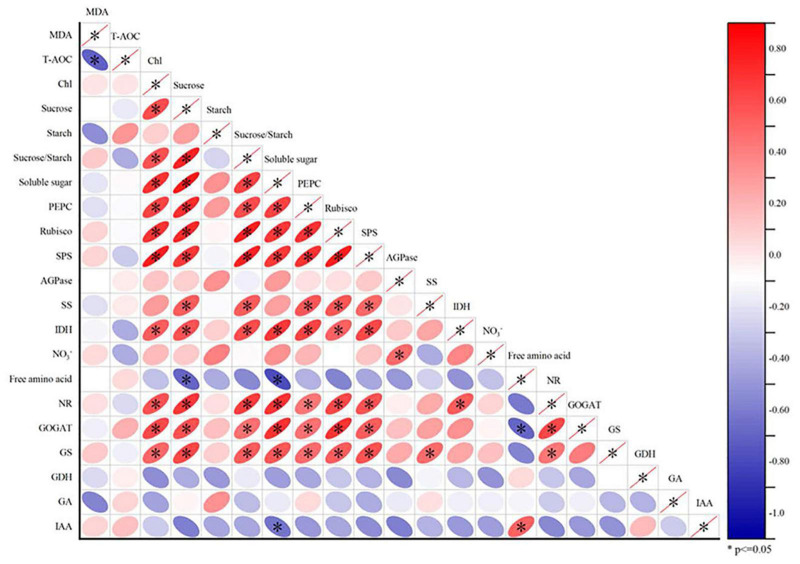
Correlation analysis of maize leaf oxidative stress (MDA, T-AOC), carbon assimilation (Chl, sucrose, starch, sucrose/starch ratio, soluble sugar, PEPC, Rubisco, SPS, AGPase, and SS), organic acid metabolism (IDH), nitrogen metabolism (NO_3_^–^, free amino acid, NR, GOGAT, GS, and GDH) and endogenous hormones (IAA and GA). The color of the ellipse in each small square represents the degree of correlation between the two indicators, and the orientation of the ellipse indicates a correlation is positive or negative. *P < 0.05 indicates statistically significant correlations.

We performed a Pearson correlation analysis (Figure 4) of the indicators at V6 to clarify the interaction between different indicators and elucidate changes in maize growth in response to PGRs.

There was a strong positive correlation between carbon and nitrogen metabolism activities (including carbon metabolism enzymes PEPC, Rubisco and SPS and nitrogen metabolism enzymes NR, GOGAT, and GS) in response to the application of PGRs. This interaction occurs not only between carbon metabolites and sucrose (sucrose-to-starch ratio and sucrose, soluble sugars, and sucrose contents), but also between carbon metabolites and carbon and nitrogen metabolizing enzymes (sucrose and PEPC, Rubisco, SPS, SS, IDH, NR, GOGAT, and GS). In addition, we also observed a negative correlation between MDA and T-AOC. There were weak correlations among the contents of FAA and GDH, GA and IAA, but a significant positive correlation was observed between IAA and FAA contents.

### Principal Component Analysis and Partial Least Squares Discriminant Analysis

To more comprehensively reveal the biological variation and metabolic activity characteristics of maize leaves treated with different PGRs at V6, but not at V10 and V14, we conducted principal component analysis (PCA) and partial least squares discriminant analysis (PLS-DA) with MetaboAnalyst 5.0^2^ (Supplementary Figure 5).

Before performing multivariate data analysis, the following operations were performed: sample normalization (normalization based on summation), data transformation (log_10_ transformation) and data scaling (Pareto scaling) (Supplementary Figure 5A). In the PCA model, PC1 explained 50.2% of the variance in the data, and PC2 explained another 15.8%. The score scatter plot clearly showed an obvious separation between the control group and the PGR treatment groups (Supplementary Figure 5B), and the loading of the scatter plot indicated that most of the indicators (except FAA, IAA, and GDH) in carbon and nitrogen metabolic enzyme activities were responsible for the differentiation of the treatment groups based on their individual characteristics (Supplementary Figure 5C). However, the PCA model is based on the grouping with the highest variation in the sample, which ignores the distinct effects of the various PGRs. Therefore, we chose to use PLS-DA, a supervised multivariate analysis, to test separation between treatments. Data preprocessing was consistent with PCA. The reliability of the model was verified by 100 permutation tests and cross-validation, which was crucial for subsequent analysis (Supplementary Figures 5D,E). In PLS-DA, PC1 and PC2 explained 27.6 and 35% of the variance in the data, respectively (Supplementary Figure 5F). The score plot divided all treatments into three regions: (i) Control group, (ii) trehalose treatment, (iii) chitosan, humic acid, and gamma-aminobutyric acid treatments combined. To further identify the variables underlying the differences in partitions, we utilized variable importance (VIP) in projections. VIP describes the importance of each variable to the model, with its partition contribution, and variables for which VIP > 1.5 and *P* < 0.05 (one-way ANOVA) were considered significant. Based on the above analysis, we found that starch content (VIP = 2.30, *P* < 0.01) and sucrose content (VIP = 1.83, *P* < 0.01) were the only two significant indicators in the PLS-DA model (Supplementary Figure 5G).

### Reverse Transcription Quantitative PCR

A more in-depth method was used to analyze the expression changes of key genes in the carbon and nitrogen metabolism pathway by RT-PCR at V6 (Supplementary Figure 6). Compared with control conditions, *rca1*, *fgs1*, *sps1*, *pep1* and *NR* were significantly up-regulated by trehalose and chitosan treatment by 0.88 and 0.9, 1.67, and 0.7, 1.27 and 10.7 and 1.85 and 1.1 times, respectively. In addition, trehalose treatment also up-regulated *GRMZM2G057910* expression. However, most gene expression changes were not significant in plants treated with humic acid and gamma-aminobutyric acid, except that *fgs1* and *sps1* were up-regulated by humic acid treatment by 0.32 and 0.54 times, respectively, and *sps1* and *pep1* were up-regulated by gamma-aminobutyric acid treatment by 0.67 and 1.6 times, respectively. Moreover, all PGR treatments significantly down-regulated the expression of *gdh1*.

**FIGURE 5:**
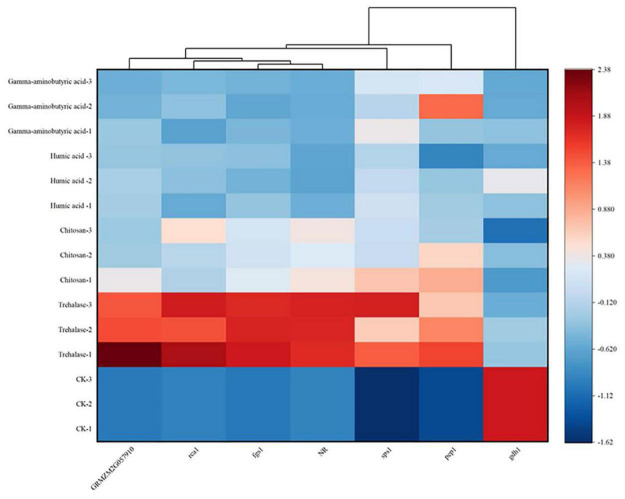
Heat map of differentially expressed genes in maize leaves treated with plant growth regulators (PGRs) at the six-leaf stage of vegetative growth (V6) stage. Columns and rows in the heatmap represent genes and samples, respectively. The sample names are displayed to the left of the heatmap. The color scale indicates fold changes in gene expression. Blue and red indicate decreases and increases in transcript abundance, respectively. A cluster analysis of differentially expressed genes is shown above the figure. Heat maps were drawn with Origin software (2021b) based on normalized *Z*-scores.

To better reveal the trend in gene expression under PGR treatment, a heat map was rendered (Figure 5). The heat map reveals a clear difference between the effects of trehalose and other PGRs. This difference is reflected in the darker red color indicating a higher change in gene expression, and although other PGRs were also significantly up-regulated, the degree of up-regulation was weaker than that observed under trehalose treatment.

## Discussion

### Plant Oxidative Stress Levels and Growth Status

Generally, plants under stress exhibit an increase in the activity of antioxidant enzymes and the content of osmotic regulators, thereby increasing their resistance to adverse conditions (Reddy et al., 2004; Zulfiqar and Ashraf, 2022). MDA is generally regarded as a biomarker of disruptions of homeostasis in plants (Sofo et al., 2004). T-AOC is an indicator of total antioxidant capacity of plants. However, we did not detect significant changes between them, indicating that plant oxidative stress levels were not changed or were changed very little by PGR treatments (Table 1).

Chlorophyll is one of the most important parameters determining the photosynthesis and yield formation of C_4_ crops (Guidi et al., 2019). Supplementary Figure 1 shows that the chlorophyll content of the trehalose treatment group was increased significantly by 11.6%, compared with the control conditions at V6. This may be explained by trehalose treatment protecting the integrity of chromosomal and chloroplast membrane structures in maize leaves (Islam and Mohammad, 2021), thereby improving the absorption and utilization of light energy by plants. This was consistent with the observed increase in biomass of maize treated with trehalose (Figure 1). However, we also observed that gamma-aminobutyric acid treatment significantly increased plant biomass, although chlorophyll content did not change significantly. This pair of contrasting results suggested that changes in chlorophyll content might not be a comprehensive indicator of stress response induced by PGRs. The root-to-shoot ratio (Figure 1C) is an important parameter of plant growth coordination, and only trehalose treatment significantly increased the root-to-shoot ratio of maize at V6 and V10. In contrast, gamma-aminobutyric acid did not significantly increase the root-to-shoot ratio at V6, but significantly increased the root-to-shoot ratio at V10. According to Lin et al. (2017) exogenous trehalose increased the root-shoot ratio of tobacco, especially under low nitrogen conditions. These results suggest that more complex metabolic activity might be involved in the regulation of maize growth and development.

### Comprehensive Analysis of Carbon and Nitrogen Metabolic Activities and Endogenous Hormones

Carbon and nitrogen are the most abundant elements in cells and have irreplaceable roles in regulating organ development, material metabolism and grain formation (Xu et al., 2020). Carbon assimilation provides both the carbon skeleton and energy for nitrogen metabolism.

Photosynthesis is the basis of dry matter production and is limited by the intensity of the carbon metabolism in the internal environment of plants (Stitt, 1986). Carbon metabolic intensity is limited by two factors: (1) The accumulation and transport capacity of photosynthates; (2) the content or activity of carbon metabolic enzymes. Sucrose is the final product of photosynthesis and the most abundant soluble storage carbohydrate in most plants (Deng et al., 2019). SPS and SS are the rate-limiting enzymes in sucrose synthesis and the key enzymes in the conversion of sucrose to starch, respectively. In addition, Rubisco is a key enzyme in the Calvin cycle, catalyzing the fixation of net CO_2_ in all photosynthetic organisms (Atkinson et al., 2020). Compared with the control conditions, trehalose treatment significantly increased sucrose accumulation at V6 and V10, which was related to the increase of SPS, SS and Rubisco enzyme activities; correlation analysis revealed significant positive correlations between them (Figure 2A and Supplementary Figures 2A–C). Yu et al. (2021) found that a short-term treatment with 100 mM trehalose resulted in a large drop in SS and a considerable increase in SPS, and that the activities of SS and SPS were regulated by SnRK. Our results demonstrated a strong positive correlation between SS and sucrose synthesis, despite the fact that SS is responsible for sucrose decomposition in general. The following are a few explanations for this discrepancy: (1) The trehalose concentration used in this investigation was 100 mg/L (< 100 mM). (2) SnRK1 regulates the breakdown of SS, which is a passive process controlled by sucrose levels. During periods of excess carbohydrate synthesis, leaves divert hexose to starch synthesis to maintain a high level of light energy utilization and avoid photoinhibition (McKinley et al., 2018). AGPase regulates the first rate-limiting step in starch biosynthesis (Yoon et al., 2021). Compared with the control conditions, the starch content under chitosan, humic acid and gamma-aminobutyric acid treatments was significantly increased at V6. In contrast, the starch content under trehalose treatment did not change significantly at V6 (Figure 2B). This was basically consistent with the trend in AGPase activity, although starch content and AGPase activity were not positively correlated (Figure 4 and Supplementary Figure 2D). The sucrose-to-starch ratio is considered an important factor regulating plant growth and yield formation (Li et al., 2018). In our experiment, the sucrose-to-starch ratio was significantly increased by trehalose and chitosan (Figure 2D). Pearson correlation analysis revealed that the sucrose-to-starch ratio was positively regulated by sucrose, but not starch; this was consistent with the observed trends in sucrose content and the sucrose-to-starch ratio (Figures 2A,D, 4). PCA and PLS-DA joint analysis revealed the accumulation of sugar and starch was an important control target of PGR, and sucrose and starch accumulation treatment group could be divided into three parts: (1) The control conditions, with low starch and sucrose; (2) the trehalose treatment, with high sugar and low starch; (3) other PGR treatment, with low sugar and high starch (Supplementary Figure 5). The sucrose availability signal is regulated by trehalose-6-phosphate (Tre6P), and high levels of Tre6P inhibit the mobilization of starch, thereby regulating the contents of sucrose and starch (Hamilton et al., 2021). Exogenous trehalose may increase sucrose synthesis and decrease starch synthesis by promoting Tre-6P (positive feedback), in contrast with the negative feedback caused by the accumulation of endogenous trehalose. Plants utilize sucrose during the day and store starch for metabolism at night (Liu et al., 2019), which may reflect a rapid remobilization of photosynthates, which plays an important role in promoting plant growth under trehalose treatment.

In particular, PEPC catalyzes the formation of oxaloacetate from phosphoenolpyruvate (PEP) in the presence of Mg^2+^, and IDH regulates the synthesis of organic acid anions, providing 2-oxoglutaric acid (2-OG) for ammonia assimilation (Cai et al., 2022). The activity of PEPC was significantly increased under all PGR treatments at V6. Among treatments, only trehalose significantly increased the activity of IDH at V6 (Supplementary Figures 2E,F). Changes in IDH activity may result in a drop in NAD and NADPH content, causing a disruption in cell redox levels (Foyer et al., 2011). Thus, trehalose is the only one of the four PGRs that regulates tricarboxylic acid cycle flux throughout the whole growth cycle (V6, V10, and V14). Furthermore, the changes in carbon assimilation activities induced by PGRs may further affect primary nitrogen metabolism. NO_3_^–^, as the major form of nitrogen available to most higher plants, is reduced to ammonium salts by NR, which is assimilated into amino acids through the GDH and GS-GOGAT pathways. NO_3_^–^ is catalyzed by NR, which is the rate-limiting step in nitrogen assimilation (Han et al., 2022). In general, the application of PGRs did not significantly change NO_3_^–^ content, except humic acid did significantly increase its accumulation at V6. However, the activities of NR, GOGAT, and GS were significantly increased by trehalose. Compared with trehalose treatment, chitosan only significantly increased the activities of NR and GOGAT (Figure 3A and Supplementary Figures 3A–C). Ozfidan-Konakci et al. (2018) reported that humic acid enhanced nitrogen uptake by plants and promoted cell elongation and division. Lin et al. (2017) described the promoting effect of trehalose on nitrogen metabolism. Correlation analysis revealed a significant positive correlation between the activity levels of carbon metabolism enzymes (PEPC, Rubisco, SPS, and SS) and nitrogen metabolism enzymes (NR, GOGAT, and GS) (Figure 4). The accumulation of plant biomass and the synthesis of carbon metabolites were also further improved (Figures 1, 2 and Supplementary Figure 2). Therefore, the application of trehalose and chitosan appears to accelerate the transformation and assimilation of NO_3_^–^. The concentration of FAA is regulated by both plant growth and nitrogen accumulation status (Atilio and Causin, 1996). Chitosan significantly reduced the accumulation of FAA in leaves, but there was no significant difference in leaf FAA content under other PGR treatments at V6 (Figure 3B). There was a negative correlation between FAA and sucrose and soluble sugar accumulation (Figure 4). However, the importance of increased sucrose for improved plant growth is demonstrated by the results (Figures 1, 2, 4 and Supplementary Figure 5). Accordingly, this might reveal why the effect of the chitosan treatment was weaker than that of the trehalose in terms of carbon and nitrogen metabolic intensity. GDH mainly exists in mitochondria and participates in detoxification of ammonium and replenishment of glutamate pools in plants (Ashraf et al., 2018). All PGR treatments tended to inhibit GDH activity, and chitosan had the most significant inhibition effect at V6 (Supplementary Figure 3D). This might be caused by chitosan enhancing the flow of ammonium into the GS-GOGAT cycle by inhibiting excessive accumulation of glutamate, as high concentrations of glutamate can inhibit root growth in rice and cucumber (Kim et al., 2010). GDH participates in the regulation of ammonium ion concentration and limits ammonium poisoning. However, we did not detect the generation of oxidative stress (Table 1). Therefore, GDH and FAA may tend to be inhibited under normal conditions under the application of PGRs, which may further enhance nitrogen metabolic activity.

Changes in endogenous hormones are another important strategy driving plant growth. Plant hormones regulate all developmental activities (Lin et al., 2020). IAA and GA control different processes, but cooperate in the formation and development of source and sink organs (Duan et al., 2018; Siddiqui et al., 2022). At the Overall level, compared with the control conditions, the application of PGRs did not significantly change the contents of GA and IAA. At the individual level, there were significant differences in the application of different PGR treatments compared with control conditions. Generally, the effects of PGRs on GA were mainly concentrated at V6 and V10, while the effects on IAA were mainly concentrated at V10 and V14 (Supplementary Figure 4). Although trehalose and chitosan significantly upregulated carbon and nitrogen metabolism, they did not significantly change the content of GA and IAA in leaves. In addition, no significant interaction between carbon and nitrogen metabolic activities and hormone levels was revealed by the Pearson correlation analysis (Figure 4).

Thus, PGR treatment had a short-term promoting effect on maize metabolic activity until V6 (0–12 days), and a long-term cumulative effect of metabolites associated with maize growth until V10 (13–28 days). Over the longer time period until V14 (29–46 days), no significant changes in carbon and nitrogen metabolic activity were observed. This may be explained by the effect of the PGR treatments being diminished or by the metabolic activity being converted into a potential capacity for improvement of grain yield or quality at maturity. One important strategy for using PGRs to improve plant growth status is through regulation of the intensity of carbon and nitrogen metabolic activity, rather than by changing endogenous hormone levels.

### Comprehensive Analysis of Key Genes in the Carbon and Nitrogen Metabolism Pathway

Gene expression levels in key metabolic pathways are associated with changes in enzyme activity or content (Supplementary Figure 6). *GRMZM2G057910* encodes IDH, a key enzyme in the tricarboxylic acid cycle that is responsible for catalyzing the oxidative decarboxylation of isocitrate (Vega, 2020). Compared with the control conditions, the expression level of *GRMZM2G057910* in the trehalose treatment group was significantly up-regulated. The gene *rca1* encodes Rubisco in plants (Erdal, 2019), and activation of Rubisco enzymes is associated with high net photosynthesis. Compared with the control conditions, the *rca1* expression levels in the trehalose and chitosan treatment groups were significantly increased. The gene *sps1* controls the synthesis of SPS enzymes, thereby actively participating in the synthesis and distribution of carbon assimilation products. The application of PGRs significantly up-regulated *sps1* expression. Compared with the control conditions, except humic acid, other PGR treatments significantly up-regulated the expression level of *pep1*, the gene that encodes PEPC. The up-regulation of carbon metabolism function genes increased the levels of related enzymes in the synthetic pathways, resulting in a net accumulation of carbon metabolites (sucrose, starch and soluble sugar) (Figure 2), which provided sufficient material and energy sources for plant biological activities. NR is encoded by the *NR* gene and involved in the synthesis of amino acids and proteins through the GS/GOGAT cycle and GDH pathway. Additionally, its activity and content can affect the accumulation of ammonium. Trehalose and chitosan significantly up-regulated the expression of *NR* genes. The gene *fgs1* encodes a protein related to GOGAT synthesis. Compared with the control conditions, the expression of *fgs1* in the trehalose, chitosan and humic acid treatment groups was significantly increased. GDH is encoded by *gdh1* and has a dual function in regulating the concentration of ammonium ions in plants. All PGR treatments significantly decreased the expression of *gdh1*. The expression patterns of nitrogen metabolism function genes were basically consistent with the changes in enzyme activities (Supplementary Figure 3). The increase in nitrogen metabolism enzyme activity indicates the enhancement of nitrogen absorption and assimilation ability. Therefore, our results revealed the positive effects of PGR application on functional genes associated with carbon and nitrogen metabolism. Compared with humic acid and gamma-aminobutyric acid, trehalose and chitosan had the most significant promoting effects. Our results were consistent with the previously reported effects of chitosan and trehalose enhancing carbon and nitrogen assimilation activities (Li et al., 2022). The heat map in Figure 5 shows the differential expression of carbon and nitrogen metabolism genes, clarifying the changes induced by PGR treatments more intuitively. Trehalose treatment increased the expression of more functional genes than did the chitosan treatment. In addition, cluster analysis results also indicated that the expression of *gdh1* was different from that of other genes, which might be owing to GDH supplying 2-ketoglutarate to carbon-limited tissues to participate in carbon cycling, rather than the reassimilation of excess ammonium (Dubois et al., 2003). However, carbon limitation did not occur under the PGR treatments. On the contrary, a rapid nitrogen assimilation process may lead to the occurrence of ammonium toxicity.

RT-qPCR and various analytical methods clarified the effects of PGRs on maize growth. The differential regulation of carbon and nitrogen metabolism by trehalose, chitosan, humic acid and gamma-aminobutyric acid is a critical factor affecting the morphology of maize. Compared with chitosan, humic acid and gamma-aminobutyric acid treatments, trehalose treatment showed a stronger promoting effect on maize growth and development. However, there was a time limit to this enhancement. Nonetheless, this study fills a gap in the present understanding of the effects of different PGRs on maize growth and has applied value in enhancing agricultural production. In addition, environmental factors such as light, rainfall and soil texture, among others, brought about by different geographical locations and climate changes may limit the application of PGRs, so a more comprehensive analysis is merited.

## Data Availability Statement

The original contributions presented in this study are included in the article/Supplementary Material, further inquiries can be directed to the corresponding author/s.

## Author Contributions

TG provided help and advice on sample processing. WZ provided guidance and support on the experimental design. BL completed the supplemental experimental content and wrote the manuscript. All authors contributed to the article and approved the submitted version.

## Conflict of Interest

The authors declare that the research was conducted in the absence of any commercial or financial relationships that could be construed as a potential conflict of interest.

## Publisher’s Note

All claims expressed in this article are solely those of the authors and do not necessarily represent those of their affiliated organizations, or those of the publisher, the editors and the reviewers. Any product that may be evaluated in this article, or claim that may be made by its manufacturer, is not guaranteed or endorsed by the publisher.

## Footnotes

1
https://www.ncbi.nlm.nih.gov

2
https://www.metaboanalyst.ca
