# Supplementary material for: Trehalose Outperforms Chitosan, Humic Acid and Gamma-Aminobutyric Acid in Promoting the Growth of Field Maize
Source: Front Plant Sci. 2022 Jun 14;13:889615. doi: 10.3389/fpls.2022.889615 (PMC9237543; doi:10.3389/fpls.2022.889615)
Supplement: Supplementary file 1 [file Data_Sheet_1.PDF]

Supplementary information:

Evaluation: The effects of different plant growth regulators on maize growth and metabolism

Bingyan Li[a], Tengfei Guo[b], and Wei Zhou[a*]

[a] *Institute of Agricultural Resources and Regional Planning, Chinese Academy of Agricultural Sciences, Beijing 100081, China*

[b] *Institution of Plant Nutrition and Environmental Resources, Henan Academy of Agricultural Sciences, Zhengzhou, 450002, PR, China.*

82101201117@caas.cn

guotengfei@hnagri.org.cn

Corresponding Author:

Wei Zhou: *zhouwei02@caas.cn*

Table S1. Amplification primers for RT-qPCR.

Genes	Direction	Primer sequences (5'-3')
GRMZM2G057910—IDH	F	AAACTCGAGGCTGCTTGCGTTGAGA
	R	ATAATTAGCTTGCATCGAAACTGCGG
rca1—Rubisco	F	GCAAAGGCCAGGGAAAATCG
	R	ATGTTCATCAGGGTGGCGTT
gdh1—GDH	F	GATGTAGCCCTGGAGACC
	R	TCCCTGTATGACGAAACG
fgs1—GOGAT	F	GCAATCCTGAACCTTTCTCTG
	R	GATGCTCGCAGTCTCATGTT
sps1—SPS	F	CTACAGCAGCGGGAACG
	R	TCGCCACAGACGAGGGT
pep1—PEPC	F	TTTCAGACGAGTCATAATGTCATCAC
	R	CTTCTCAGGTGGTCTGCATCATAT
NR—NR	F	GACGCCATCCACTACGACATG
	R	ACCTTGACGAGGAGGTCCAAGT
actb1—β-actin	F	TGTTTCGCCTGAAGATCACCCTGTG
	R	TGAACCTTTCTGACCCAATGGTGATGA

F, forward; R, reverse. IDH: isocitrate dehydrogenase, Rubisco: 1,5-diphosphate ribulose carboxylase, GDH: glutamate dehydrogenase, GOGAT: glutamine synthase, SPS: sucrose phosphate synthase, PEPC: Phosphoenolpyruvate carboxylase, NR: nitrate reductase, β-actin: Actin.

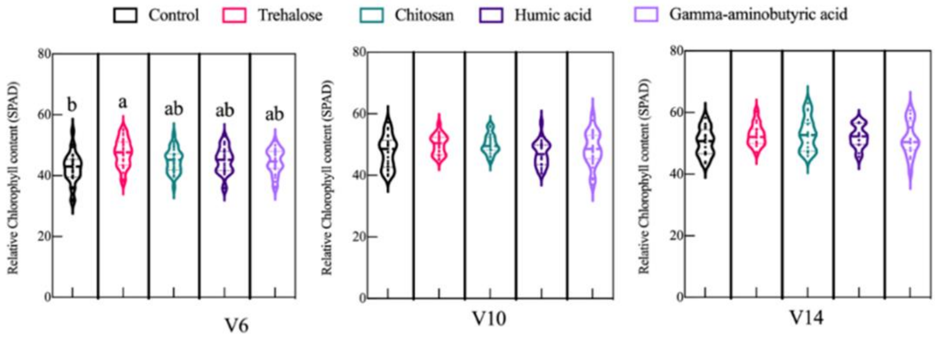

Figure S1. The effect of plant growth regulators (PGRs) on the chlorophyll content of maize at different growth stages. The black dotted horizontal line in the figure represents the median, and the relative width of the curve represents the density of the data. Statistical analysis was used to compare differences in means between PGR treatments shown in different colors. Within each stage, treatments labeled with different lowercase letters were significantly different ($P < 0.05$), and the lack of such letters indicates the lack of a significant difference. V6, V10 and V14 represent the 6-, 10- and 14-leaf stages of vegetative growth, respectively. $n = 20$.

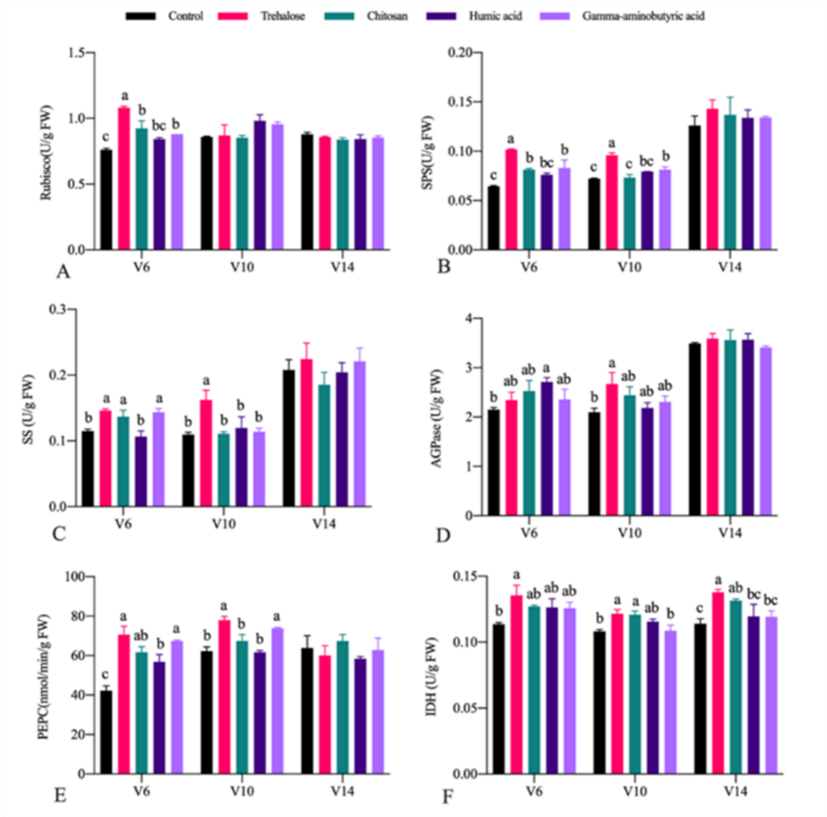

Figure S2. Effects of plant growth regulators (PGRs) on the activities of carbon metabolism enzymes in maize at different growth stages. Statistical analysis was used to compare means of the PGR treatments shown in different colors; significantly different treatments within each stage are labeled with different lowercase letters ($P < 0.05$), and the lack of such letters indicates the lack of significant differences. V6, V10 and V14 represent the 6-, 10- and 14-leaf stages of vegetative growth, respectively. FW, fresh weight; $n = 3$.

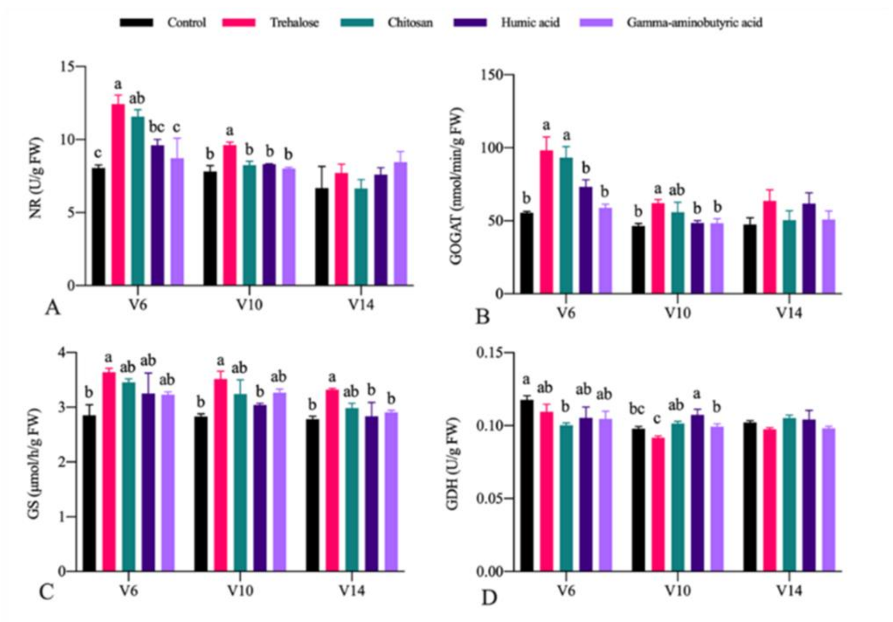

Figure S3. Effects of plant growth regulators (PGRs) on the activities of nitrogen-metabolizing enzymes at different growth stages of maize. Statistical analysis was conducted to compare means of the PGR treatments shown in different colors; treatments labeled with different lowercase letters within each stage were significantly different ($P < 0.05$), and the lack of lowercase letters indicates there were no significant differences. V6, V10 and V14 represent the 6-, 10- and 14-leaf stages of vegetative growth, respectively. FW, fresh weight; $n = 3$.

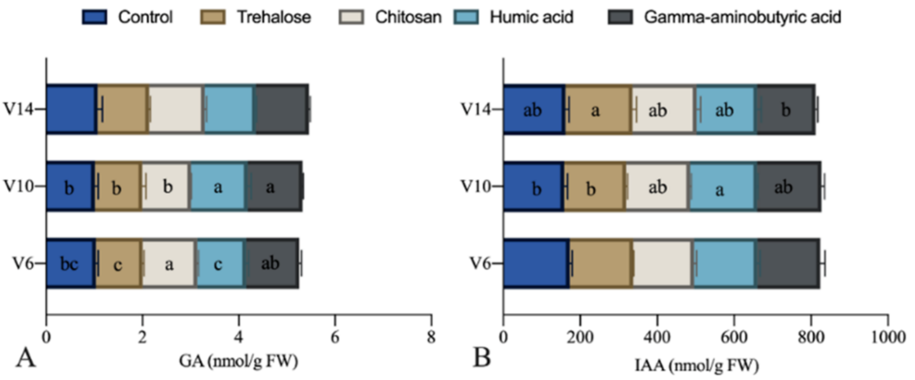

Figure S4. Effects of PGRs on endogenous hormone levels in maize at different growth stages. Statistical analysis occurred in PGRs treatments with different colors in the same growth period. Significant results are marked with lowercase letters on the figure, and no mark indicate no significant difference. V6, V10 and V14 represent the 6-leaf stage, 10-leaf stage and 14-leaf stage of vegetative growth, respectively. significant level (P<0.05). FW, fresh weight.

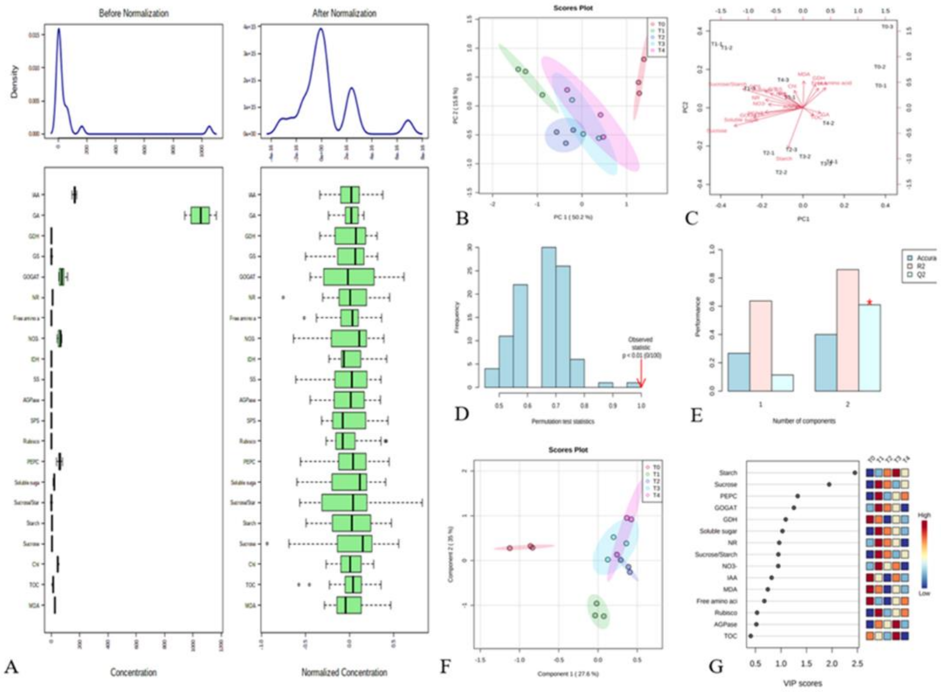

Figure S5. Principal component analysis (PCA) and partial least squares discriminant analysis (PLS-DA) of oxidative stress (MDA, TOC), carbon assimilation (Chl, sucrose, starch, sucrose/starch ratio, soluble sugar, PEPC, Rubisco, SPS, AGPase and SS), organic acid metabolism (IDH), nitrogen metabolism (NO_3^-, FAA, NR, GOGAT, GS and GDH) and endogenous hormones (IAA and GA) in maize leaves under different biomodulator treatments. (A) Data normalization plot. (B) PCA score plot. (C) PCA loading plot. (D) 100 permutations test statistics of PLS-DA. (D) Cross-validation of PLS-DA. (F) PLS-DA score plot. (E) VIP scores of PLS-DA. T0, T1, T2, T3, and T4 represent the control, trehalose, chitosan, humic acid and γ-aminobutyric acid treatments, respectively.

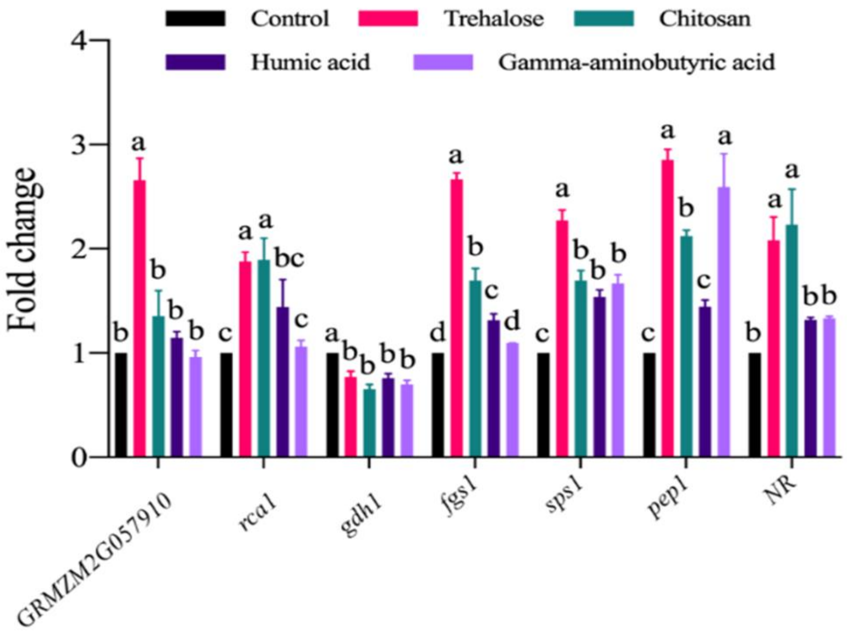

Figure S6. The relative expression levels of PGRs on the differential genes of carbon and nitrogen metabolism in maize V6 stage were detected by RT-qPCR. Values represent the mean ± SE; n = 5.
